# A new species of louse fly, *Ornithomya* Latreille, 1802 (Diptera: Hippoboscidae), from the Russian far East

**DOI:** 10.1016/j.ijppaw.2025.101111

**Published:** 2025-07-01

**Authors:** Aleksandra Yatsuk, Emilia Nartshuk, Tatiana Triseleva, Valeriy Shokhrin, Oleg Tolstenkov, Andrey Safonkin, Alexandr Matyukhin

**Affiliations:** a A.N. Severtsov Institute of Ecology and Evolution, Russian Academy of Sciences, 33 Leninskiy Av., 119071, Moscow, Russia; b Zoological Institute of the Russian Academy of Sciences, Universitetskaya emb. 1, Saint Petersburg, 199034, Russia; c United administration of the Lasovsky State Nature Reserve named after L.G. Kaplanov and “Zov Tigra” National Park, 56 Tsentralnaya St., Lazo, Primorskiy Territory, 692980, Russia; d Michael Sars Center, University of Bergen, Bergen, Norway

**Keywords:** Diptera, Hippoboscidae, Louse flies, New species, *Ornithomya*, Russia, Russian far east

## Abstract

Louse flies (Diptera: Hippoboscidae) are specialized ectoparasites of birds, influencing host health and potentially acting as vectors of pathogens. A new species of the genus *Ornithomya* Latreille, 1802 (Diptera: Hippoboscidae), *Ornithomya panovi***sp. n.**, is described from specimens collected in Lazovsky Nature Reserve (Russia). *O. panovi***sp. n.** belongs to the *avicularia* species-group and is distinguished from other known Ornithomya species inhabiting Russia and Japan by several morphological features: reduction of tergite 4, wing length, ratio of costal vein sections between junctions R1 and R2+3 and between junctions R2+3 and R4+5 and arrangement of wing microtrichia as well as by genetic distances. This discovery expands our knowledge of the region's parasite biodiversity and highlights the need for continued faunistic surveys in the Russian Far East.

## Highlights

• A new louse fly species is described from the Russian Far East.
• The species is clearly distinguished by a unique reduction of tergite 4.
• Combined morphological and molecular data confirm the species' taxonomic status.
• *Ornithomya panovi* sp. n. belongs to the avicularia species-group.

## Graphical abstract

**Figure:**
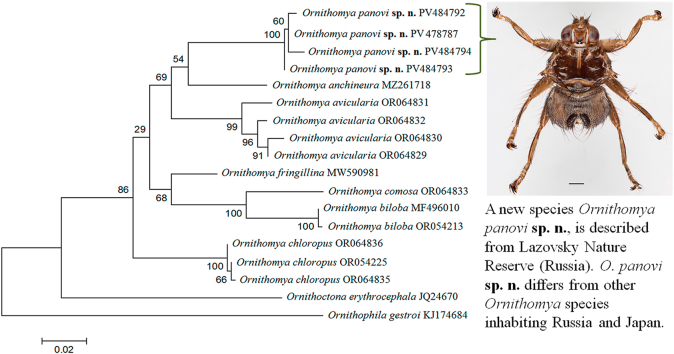

## 1 Introduction

Parasitic insects play crucial roles in ecosystems by influencing host health, behavior, and population dynamics, yet many remain understudied, particularly in remote regions of Northeast Eurasia. The ectoparasite family Hippoboscidae Samouelle, 1819 includes over 200 fly species (Dick, 2006; Oboňa et al., 2019). These flies are hematophagous ectoparasites that feed on the blood of mammals and birds (Hutson, 1984). They serve as vectors for numerous pathogens, posing epidemiological risks (Bequaert, 1954; Doszhanov, 1980, 2003; Ganez et al., 2004; Farajollahi et al., 2005; Khametova et al., 2018; Peña-Espinoza et al., 2023; Wawman, 2023), and are able to transport phoretic mites (Fain, 1965a, b; Hill et al., 1967; Philips and Fain, 1991) and also feather lice (de Moya, 2019; Lee et al., 2022).

One of the largest genera, widely represented in the Palearctic, is the genus *Ornithomya* Latreille, 1802. All *Ornithomya* species exclusively parasitize birds (Maa, 1969; Doszhanov, 1980, 2003) and predominantly inhabit the temperate and middle latitudes of the Old World (Hutson, 1984). Currently this genus includes 33 living species (Dick, 2006; Nartshuk et al., 2022, 2024; Yatsuk et al., 2023, 2024a, 2024b; Matyukhin et al., 2023) and one fossil species (Maa, 1966). This species are divided into five groups, based on their morphology (Maa, 1963; Dick, 2006).

To date, 12 species of *Ornithomya* have been recorded within the territory of Russia and the former USSR: *O. avicularia avicularia* L., 1758, *O. biloba* Dufour, 1827; Doszhanov, 1980, 2003), *O. candida* Maa (1967); Yatsuk et al. (2024d), *O. chloropus* Bergroth, 1901, *O. comosa* Austen, 1930, *O. fringillina* Curtis, 1836; Doszhanov, 1980, 2003), *O. strigilis* Nartshuk, Yatsuk et Matyukhin, 2022; Nartshuk et al. (2022), *O. triselevae* Matyukhin, Yatsuk et Nartshuk, 2023; Matyukhin et al. (2023), *O. krivolutskii* Yatsuk, Matyukhin et Nartshuk, 2023; Yatsuk et al. (2023), *O. nazarovi* Yatsuk, Matyukhin et Nartshuk et al., 2024; Yatsuk et al. (2024a), *O. delichoni* Yatsuk, Matyukhin et Nartshuk et al., 2024; Nartshuk et al. (2024), *O. helvipennis* Yatsuk, Nartshuk et Matyukhin et al., 2023; Yatsuk et al. (2024b).

Some Ornithomya species exhibit extensive morphological variability, for example, in the number of scutellar setae, complicating species identification using traditional morphology alone (Doszhanov, 1980, 2003; Maa, 1967, 1969). As a result, molecular methods are increasingly employed to clarify taxonomy and resolve cryptic species boundaries within Hippoboscidae (Meiβner et al., 2020; Petersen et al., 2007a, Petersen et al., 2007b; Yatsuk et al., 2024c, 2024d).

Meiβner et al. (2020) reported molecular data on Ornithomya species from the Amur region in Russia, some of which had not been previously identified. The aim of this study is to describe a new species of *Ornithomya* collected from birds in the Russian Far East. This study contributes to filling existing gaps in our knowledge of louse fly biodiversity in temperate Asia.

## 2 Material and methods

The flies were collected during bird ringing in the Lazovsky Nature Reserve in the south of the Primorskiy Territory of Russia. The territory of the reserve is a typical mid-mountain area with average hill heights of 600–900 m above sea level. Forests cover 96 % of the territory. The climate is monsoonal, which is manifested in a pronounced change in wind directions in summer and winter. There are two climatic zones in the territory of the reserve: coastal and continental. The coldest month is January, with an average temperature of –8 °C on the coast and −13 °C in the continental part. The warmest month is August, with an average temperature of about +19 °C.

During the study in summer 2023, 111 birds from 25 species were examined. Among them 52 birds from 19 species were infested with flies. Of these birds one blue-and-white flycatcher (*Cyanoptila cyanomelana* (Temminck, 1829)), six grey-headed buntings (*Osyris spodocephalus* (Pallas, 1776)), ten yellow-rumped flycatchers (*Ficedula zanthopygia* (Hay, 1845)) and fourteen eurasian nuthatchs (*Sitta europaea* Linnaeus, 1758), were caught. Flies were collected from one *C. cyanomelana*, five *Os. spodocephalus*, six *F. zanthopygia,* and twelve *S. europaea*.

The louse fly material was preserved in 96 % ethanol. Morphological terminology follows Hutson (1984). Body length was measured using the standard method for Hippoboscidae (Doszhanov, 1980). The combined head and thorax length was measured from the anterior margin of the head, excluding the antennae, to the posterior margin of the scutellum. We follow the interpretation of Mogi et al. (2023), who regard *Ornithomya avicularia aobatonis* (Matsumura, 1905) as a subspecies. We base our morphological comparisons on the original description by Maa (1967).

**Fig. 1:**
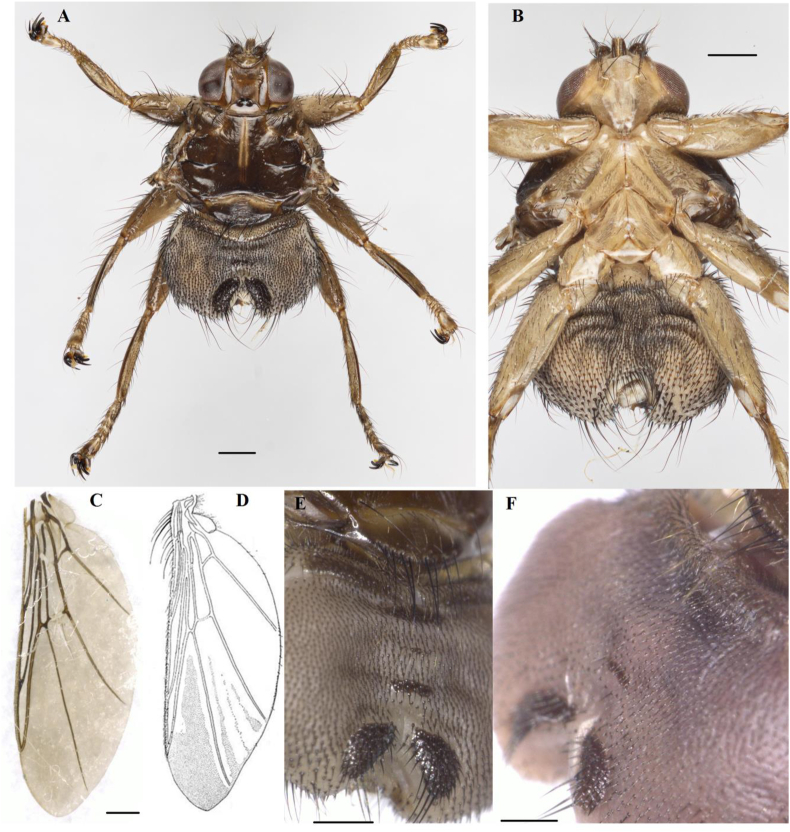
*Ornithomya panovi***sp. n.** female. A – holotype general view, dorsal side; B – holotype ventral side; C – holotype wing; D – wing drawing; E, F – examples of morphological variability of abdominal tergites, dorsal side of abdomen. Scale bars: 0.5 mm.

The morphological study was conducted using an optical microscope Keyence VHX-1000 (Japan) housed at the Joint Usage Center "Instrumental Methods in Ecology" at the Institute of Ecology and Evolution, Russian Academy of Sciences. For illustration purposes (Fig. 1) images were taken with Canon EOS 90D and Canon EOS M6 Mark II cameras with a Canon EF 100 mm/2L Macro lens, stitched and processed using Helicon Focus 7 software.

Total DNA was extracted from whole flies using Diatom-200 reagents (Isogen, Moscow) according to the manufacturer's instructions. Polymerase chain reaction (PCR) was performed using primers LCO1490 and HCO2198 (Folmer et al., 1994). Thermal cycling consisted of an initial denaturation step at 94 °C for 1 min, followed by 6 cycles of 1 min at 94 °C, 1 min at the annealing temperature of 45 °C and 1 min at 72 °C; followed by 40 cycles of 1 min at 94 °C, 1.5 min at the annealing temperature of 55 °C, and 4 cycles of 1.5 min at 72 °C; with a final extension at 72 °C for 6 min. The amplification product was purified using precipitation by ethyl alcohol solution with addition of 5M sodium acetate. Electrophoresis and reading of amplification product nucleotide sequences were carried out on an automatic ABI PRISM 3130 sequencer (Applied Biosystems, United States) using BigDye Terminator reagent kit 3.1 (Applied Biosystems). The study was conducted at the Joint Usage Center “Instrumental Methods in Ecology” at the IEE RAS.

Eleven sequences of new species were obtained. NCBI (www.ncbi.nlm.nih.gov) numbers for haplotypes are presented in Table 1. The analyzed fragment consisted of 591 base pairs (bp) of the mtDNA COI gene. Other sequences were taken from the NCBI database (Table 1). Phylogenetic relationships were analyzed using the Maximum Likelihood method with the General Time Reversible (GTR) model, assuming gamma-distributed rates among sites. The analysis included 1000 bootstrap replications and was performed using the MEGA 5.1 software package (Tamura et al., 2011). Two species, *Ornithoctona erythrocephala* (Leach, 1817) and *Ornithophila gestroi* (Róndani, 1878), were used as the outgroup.

**Table 1:** Sequence data.

Species	Sequences number	Data source	Data link
*Ornithomya anchineura* Speiser, 1905	MZ261718	NCBI	Levesque-Beaudin and Sinclair (2021)
*Ornithomya avicularia* (Linnaeus, 1758)	OR064829, OR064830, OR064831, OR064832	NCBI	Yatsuk et al. (2023)
*Ornithomya bequaerti*Maa (1969)	MZ261715	NCBI	Levesque-Beaudin and Sinclair (2021)
*Ornithomya biloba* Dufour, 1827	MF496010	NCBI	Šochová et al., 2017
*Ornithomya biloba* Dufour, 1827	OR054213	NCBI	Yatsuk et al. (2023)
*Ornithomya candida*Maa (1967)	OR064839	NCBI	Yatsuk et al. (2024d)
*Ornithomya chloropus* Bergroth, 1901	OR054225, OR064835, OR064836	NCBI	Yatsuk et al. (2023)
*Ornithomya comosa* Austen, 1930	OR064833	NCBI	Yatsuk et al. (2023)
*Ornithomya fringillina* Curtis, 1836	MW590981	NCBI	Lehikoinen et al. (2021)
*Ornithomya panovi***sp. n.**	PV478787, PV484792, PV484794, PV484793	NCBI	The current study
*Ornithomya panovi***sp. n.**	(Barcod number AEA 1495) HIPMJ020-19, HIPMJ025-19, HIPMJ026-19, HIPMJ027-19	Bold System	Meiβner et al. (2020)
*Ornithoctona erythrocephala* (Leach, 1817)	JQ246707	NCBI	Marinho et al. (2012)
*Ornithophila gestroi* (Róndani, 1878)	KJ174684	NCBI	Gutiérrez-López et al. (2015)

## 3 Results

### 3.1 Taxonomic summary

*Ornithomya panovi* Yatsuk, Matyukhin, Shokhrin, Triseleva et Nartshuk **sp. n.**

(Fig. 1)

*Type material examined:*Holotype, female: Russia, Primorskiy Terr., Lazovsky Nature Reserve, collected from blue-and-white flycatcher (*Cyanoptila cyanomelana* (Temminck, 1829)), July 17, 2023 (V.P. Shokhrin). The holotype in ethanol is deposited in the collection of the Zoological Institute of the Russian Academy of Sciences, St. Petersburg (inventory number INS_DIP_0001112).

Paratype ♀ female: Russia, Primorskiy Terr., Lazovsky Nature Reserve, collected from yellow-rumped flycatcher (*Ficedula zanthopygia* (Hay, 1845)), July 13, 2023 (V.P. Shokhrin). The paratype in ethanol is deposited in a reference collection of the A.N. Severtsov Institute of Ecology and Evolution, Russian Academy of Sciences, Moscow.

Paratypes, used to obtain molecular data.

♀ female: Russia, Primorskiy Terr., Lazovsky Nature Reserve, July 2020 (V.P. Shokhrin). NCBI number: PV484792.
♀ female: Russia, Primorskiy Terr., Lazovsky Nature Reserve, collected from wood nuthatch (*Sitta europaea* Linnaeus, 1758), July 17, 2023 (V.P. Shokhrin). NCBI number: PV484794.
♀ female: Russia, Primorskiy Terr., Lazovsky Nature Reserve, collected from wood nuthatch (Sitta europaea Linnaeus, 1758), July 18, 2023 (V.P. Shokhrin). NCBI number: PV484793.
♀ 2 females: Russia, Primorskiy Terr., Lazovsky Nature Reserve, collected from yellow-rumped flycatcher (*Ficedula zanthopygia* (Hay, 1845)) and from black-faced bunting (*Ocyris spodocephala* (Pallas, 1776)), July 17, 2023 (V.P. Shokhrin). ♀ 2 females: Russia, Primorskiy Terr., Lazovsky Nature Reserve, collected from wood nuthatch (*Sitta europaea* Linnaeus, 1758), July 15, 2023 (V.P. Shokhrin). ♀ female: Russia, Primorskiy Terr., Lazovsky Nature Reserve, collected from wood nuthatch (*Sitta europaea* Linnaeus, 1758), July 13, 2023 (V.P. Shokhrin). ♀ 2 females and ♂1 male: Russia, Primorskiy Terr., Lazovsky Nature Reserve (V.P. Shokhrin). NCBI number of their haplotype: PV478787.

*Type host:* The new species was collected on *Cyanoptila cyanomelana* (Temminck, 1829) – insectivorous bird from Southeast Asia Passeriformes.

*Etymology:* The new species is named in honor of Yevgeniy Nikolaevich Panov, doctor of biological sciences, Chief Researcher at the A. N. Severtsov Institute of Ecology and Evolution, who studied the birds of the Southern Primorye.

### 3.2 Description

Head and thorax length combined 2.5 mm.

Head with posterior part located between humeral tubercles and slightly covering anterior margin of thorax. Eye one-fourth as wide as head. Ocelli separated from each other by one width of ocellus. Inner orbits slightly widened posteriorly. Width of inner orbit almost equal to one-third of mediovertex width. Length of mediovertex equal to half of head length. 4 long orbital setae present in the center and 4 orbital setae – near antennae. Posterior margin of lunula rounded. Lunula horns located between antennae, clearly separated from lunula. Anterior margin of lunula horns notched. Palpus equal in length to second antennal segment. Antennae brown. Ventral side of head light.

Mesonotum light brown. Humeral tubercles approximately cone-shaped, protruding anterolaterally. Longitudinal, transversal and scuto-scutellar sutures clearly visible. Transversal suture interrupted in middle; longitudinal suture not reaching scuto-scutellar suture. Setae of mesonotum: 5 long and approximately 10 short humeral setae, approximately 12 black mesopleural setae, 1 of them long, 1 long and 3 short black notopleural setae, 2 long and 2 short postalar setae, 1 long and 1 short prescutellar setae. Setae of scutellum: thin short setae forming fringe on its posterior margin; 8–10 thin setae forming row on its anterior margin; under them in center of scutellum present short row of light setae; 8–10 long black setae forming transverse row along posterior margin of scutellum; row of light setae present above them. Ventral side of thorax light.

Wing length 5.0–5.3 mm. Wing with full venation, with three transverse and seven longitudinal veins. Costa interrupted before juncture with Sc; longitudinal veins R_1_, R_2+3_ and R_4+5_ connecting with costa at acute angle. Section on costa between juncture of R_1_ and R_2+3_ almost 1.3 to section between juncture of R_2+3_ and R_4+5_. The transverse vein between cells 2bc and 1m mostly unpigmented. Costa and basicosta covered with hairs. Microtrichia covering most of cell 3r, excepting small bare areas in cell base and near vien R_4+5_ and bare stripes along viens R_4+5_ and M_1+2_, and forming 3 stripes in cell 1m. Some paratypes have microtrichia stripe in cell 2m. Wing membrane light and transparent.

Legs light. Femora strong. Claws bifid. Empodium and paired pulvilli not reduced.

Abdomen covered with short setae. Tergite 1 + 2 with straight posterior margin. Tergite 3 approximately one fourth – one sixth as wide as abdomen. Tergite 5 approximately one sixth – one eighth as wide as abdomen. Tergite 4 is absent completely or reduced to a point. Tergite 6 divided into two oval sclerites, each with 2–4 strong black setae.

## 4 Discussion

### 4.1 Comparison

*O. panovi***sp. n.** differs from all other species of the genus *Ornithomya* due to the almost complete reduction of tergite 4 making it easily identifiable. Additionally, other *Ornithomya* species inhabiting Russia, north of Japan and Kuril Islands differ from the new species by the following features.

– *O. avicularia avicularia* in head and thorax length combined (3.0–3.5 mm), wing length (5.5–7.0 mm) and ratio of section of costa between junctions of R_1_ and R_2+3_ to section between the junctions of R_2+3_ and R_4+5_ (two times) (Doszhanov, 1980, 2003);
– *O. avicularia aobatonis* in postoccipital seta line on ventral side of head (long), wing length (5.3–6.5 mm) and ratio of section of costa between junctions of R_1_ and R_2+3_ to section between the junctions of R_2+3_ and R_4+5_ (two times) (Maa, 1967);
‒ *O. biloba* in palpus length (palpus longer than antennae), number of prescutellar setae (4–6) and arrangement of wing microtrichia (microtrichia cover almost entirely cells 3r and 1m and almost half of cell 2m) (Hutson,1984; Doszhanov, 1980, 2003);
– *O. candida* in number of long scutellum setae (4) and ratio of section of costa between junctions of R_1_ and R_2+3_ to section between the junctions of R_2+3_ and R_4+5_ (1.5 times);
– *O. chloropus* in number of scutellar setae (6), color of ventral side of head (light with brown triangles on sides) and color of ventral side of thorax (light with brown diamond-shaped spots);
– *O. comosa* in color of ventral side of head (dark brown), number of long scutellar setae (10–12) and arrangement of wing microtrichia (microtrichia cover all wing cells);
‒ *O. fringillina* in wing length (3.5–4.5 mm), number of long scutellar setae (4) and arrangement of wing microtrichia (microtrichia form 1 stripe in cell 2m) (Doszhanov, 1980, 2003);
‒ *O. krivolutskii* in number of scutellar setae (presence of 4 black long setae above 6 strong setae row along posterior margin of scutellum), ratio of section of costa between junctions of R_1_ and R_2+3_ to section between the junctions of R_2+3_ and R_4+5_ (two times), head and thorax length combined (3 mm), wing length (4 mm) and arrangement of wing microtrichia (microtrichia cover most of cell 3r, 1m and distal part of cell 2m) (Yatsuk et al., 2023);
‒ *O. strigilis* in head and thorax length combined (4.3 mm), wing length (7.5–8 mm) and in ratio of section of costa between junctions of R_1_ and R_2+3_ to section between the junctions of R_2+3_ and R_4+5_ (two times) (Nartshuk et al., 2022);
‒ *O. triselevae* in ratio of section of costa between junctions of R_1_ and R_2+3_ to section between the junctions of R_2+3_ and R_4+5_ (two times), wing length (5.8–6.0 mm) and number of long scutellar setae (4) (Matyukhin et al., 2023).
‒ *O. nazarovi* in number of scutellar setae (6 black long setae forming along posterior margin) and arrangement of wing microtrichia (microtrichia almost completely cover cells 3r and 1m) (Yatsuk et al., 2024a)
‒ *O. delichoni* in ratio of section of costa between junctions of R_1_ and R_2+3_ to section between the junctions of R_2+3_ and R_4+5_ (two times), color of ventral side of head (brown), color of ventral side of thorax (light with brown triangles on sides) and arrangement of wing microtrichia (microtrichia covering most of cells 3r and 1m and short part of cell 2m) (Nartshuk et al., 2024)
‒ *O. helvipennis* in ratio of section of costa between junctions of R_1_ and R_2+3_ to section between the junctions of R_2+3_ and R_4+5_ (two times), arrangement of wing microtrichia (microtrichia cover all wing cells) and color of ventral side of thorax (brown) (Yatsuk et al., 2024b).

*O. panovi***sp. n.** belongs to the *avicularia* species-group along with other species *O. alpicola* Maa, 1975, *O. anchineuria* Speiser, 1905, *O. apelta* Maa (1969), *O. avicularia*, *O. bequaerti* Maa (1969), *O. candida*, *O. chloropus*, *O. fringillina*, *O. fuscipennis* Bigot, 1885, *O. gigantea* Bear and Friedberg, 1995, *O. marginalis* Maa, 1964, *O. medinalis* Maa, 1975, *O. opposita* Walker, 1849, *O. papillosa* Maa, 1964, *O. parva* Macquart, 1843; Maa (1963); Dick (2006).

### 4.2 Molecular data

Pairwise genetic distance analysis (Table 2) showed that haplotypes from Muraviovka Park (Amur Oblast, Russia), listed in BOLD Systems and attributed by Meiβner et al. (2020) to *O. avicularia aobatonis*, are nearly identical (0.3 % difference) to *O. panovi* **sp. n.** We believe this identification was a misassignment, as the morphological traits in the figure provided by Meiβner et al. (2020) correspond closely with those of *O. panovi* **sp. n.**, notably, the absence of tergite 4, a costal vein ratio of ∼1.3:1, and wing length of 5.2 mm. Within the genus *Ornithomya* the genetic distance between individual species mostly ranges from 5.5 % between *O. biloba* and *O. comosa* to 9.8 % between *O. avicularia* and *O. comosa* (Table 2). The distance between *O. panovi* **sp. n.** and its closest species *O. anchinuera* is 6.6–6.8 %, aligning with the genetic distance between other species within the studied genus and family (Petersen et al., 2007a, Petersen et al., 2007b; Yatsuk et al., 2024e).

**Table 2:** Distances between species (%).

*Ornithomya* species	*panovi* sp. n. (from Bold System)	*panovi* sp. n. (from current study)	*avicularia*	*anchineura*	*fringillina*	*biloba*	*comosa*	*chloropus*	*candida*
*panovi***sp. n.** (from current study)	0.3								
*avicularia*	7.2	7.4							
*anchineura*	6.6	6.8	6.7						
*fringillina*	7.6	7.8	6.6	6.3					
*biloba*	8.9	9.2	8.5	8.9	6.9				
*comosa*	9.5	9.7	9.8	9.5	7.1	5.5			
*chloropus*	8.0	8.2	7.5	7.7	6.3	9.5	9.4		
*candida*	7.5	7.6	6.4	6.4	2.0	6.2	7.1	6.5	
*bequaerti*	7.3	7.5	6.2	6.4	2.0	5.8	6.8	6.5	0.3

**Fig. 2:**
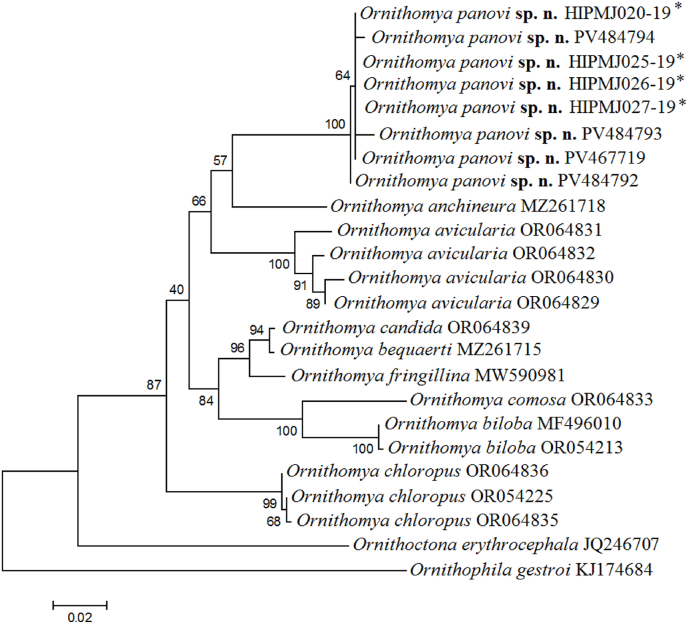
Reconstructed phylogenetic tree of the *Ornithomya* species with the representatives of the genera *Ornithophila* and *Ornithoctona* as an outgroups. Maximum likelihood bootstrap support is shown in the nods; scale bar depicts the genetic distance between haplotypes. ∗ – specimens, that were previously erroneously identified as *O. avicularia aobatonis*.

Among the 11 sequences of *O. panovi* **sp. n.** generated in this study and four sequences from BOLD Systems, we identified four haplotypes. Phylogenetic analysis (Fig. 2) indicates two species groups within the genus Ornithomya. One group includes *O. panovi* **sp. n.**, *O. avicularia*, and *O. anchinuera*, while the second group comprises *O. bequaerti*, *O. biloba*, *O. candida*, *O. comosa*, and *O. fringillina*. The species *O. chloropus* stands apart. The observed divergence of louse fly species is consistent with the phylogenetic framework proposed for the family Hippoboscidae (Yatsuk et al., 2024d). Unfortunately, molecular data are currently unavailable for several species known from the Russian Far East (*O. strigilis*, *O. triselevae*, *O. krivolutskii*, *O. nazarovi*, *O. delichoni*, and *O. helvipennis*), limiting their inclusion in the present phylogenetic analysis. Future sequencing efforts targeting these species will be essential for resolving their taxonomic placement and for achieving a more complete understanding of Ornithomya diversity in this understudied region.

Thus, the newly described species, *O. panovi* **sp. n.**, differs from all known *Ornithomya* species both morphologically and genetically. This species is likely one of the most widespread species of this genus in the Russian Far East. *O. panovi* **sp. n.** is, to our knowledge, the first described species in Ornithomya in which tergite 4 is entirely absent or reduced to a minute point.

## CRediT authorship contribution statement

**Aleksandra Yatsuk:** Writing – review & editing, Writing – original draft, Project administration, Methodology, Investigation, Data curation, Conceptualization. **Emilia Nartshuk:** Writing – review & editing, Writing – original draft, Data curation, Conceptualization. **Tatiana Triseleva:** Writing – review & editing, Writing – original draft, Methodology, Data curation. **Valeriy Shokhrin:** Writing – review & editing, Writing – original draft, Methodology. **Oleg Tolstenkov:** Writing – review & editing, Writing – original draft, Conceptualization. **Andrey Safonkin:** Writing – review & editing, Writing – original draft, Methodology. **Alexandr Matyukhin:** Writing – review & editing, Writing – original draft, Methodology, Conceptualization.

## Statements and declarations

The authors declare no competing interests.

This research was not conducted on vertebrates. Furthermore, hippoboscid flies are not a protected species and no ethical approval or research permits were required.

## Conflict of interest

The authors declare no conflict of interest.
